# Uncommon Haematological Transformation: Chronic Myeloid Leukaemia Transitioning Into Plasma Cell Leukaemia in a Single Patient

**DOI:** 10.7759/cureus.51639

**Published:** 2024-01-04

**Authors:** Mili A Jain, Sourya Acharya, Aryan S Pal, Lalit Raut

**Affiliations:** 1 Medicine, Jawaharlal Nehru Medical College, Datta Meghe Institute of Higher Education & Research (DMIHER), Wardha, IND

**Keywords:** chronic myeloid leukaemia, plasma cell leukaemia, peripheral smear, imatinib, myelocytes, leukaemia

## Abstract

A 40-year-old male patient who was a known case of chronic myeloid leukaemia (CML) was diagnosed two years back on tab imatinib 400 mg/day; he came with complaints of easy fatigability, syncopal attacks, and bone pain associated with low-grade fever for 15 days. Repeat haematological profile and a peripheral smear of the patient suggested features of plasma cell leukaemia (PCL)/plasma cell dyscrasia (PCD). A definitive treatment protocol of lenalidomide, bortezomib, and dexamethasone for PCL was prescribed to the patient. This medical case study emphasizes the rare possibility of the transformation of CML into PCL and the possible trigger of tyrosine kinase inhibitor for the same.

## Introduction

Chronic myeloid leukaemia (CML) is a myeloproliferative disorder which is characterised by the presence of Philadelphia (Ph) chromosome [1-3]. The Ph chromosome results from a translocation between the long arm of chromosome 22 and chromosome 9 [1,3]. This results in the breakpoint cluster region protein-Abelson murine leukemia viral oncogene homolog (BCR-ABL) fusion gene, which codes for a protein with abnormal tyrosine kinase activity; this plays a crucial role in determining cellular proliferation, differentiation, and survival [3]. Plasma cell dyscrasia (PCD) is a spectrum of disorders which include monoclonal gammopathies of undetermined significance (MGUS), multiple myeloma, primary amyloidosis, cryoglobulinemia, etc. [2,4]. Plasma cell leukaemia (PCL) is subcategorised within multiple myeloma, a distinctive feature being having more than 20% plasma cells in the peripheral blood [4,5]. Presenting features vary depending on the variant, primary or secondary form. Secondary varieties are the haematological transition of multiple myeloma instances, while the primary form does not show any evidence of multiple myeloma [2,5]. PCL is an aggressive form of tumour and is rarely found in the general population, and evidence also shows that tyrosine kinase inhibitors (TKI) which are the cornerstone drugs used to treat CML may induce the transformation of CML into monoclonal gammopathy [1]. This case report discusses the haematological metamorphosis happening in an individual from CML to PCL.

## Case presentation

A 40-year-old male patient had presented two years back with complaints of low-grade fever, generalized weakness, and early satiety of three-month duration. The fever was not relieved on medication, and no diurnal variation was present. On general examination, pallor was evident in the lower palpebral conjunctiva. Abdominal examination revealed massive splenomegaly, extending up to the umbilicus and non-tender on palpation with smooth edges. Mild hepatomegaly extending 2 cm below the costal margin and non-tender with a smooth surface was palpable. No other significant clinical findings were appreciated on examination.

Investigations

Haematological parameters, peripheral smear, and bone marrow aspiration suggested CML. The haematological picture revealed the following findings (Table 1).

**Table 1:** Haematological parameters of the first admission. RDW: red cell distribution width; MID#: mid-range absolute count

Parameters	Result
Haemoglobin	9.7 g/dl
Red blood cells	3.4 million/mm^3^
Haematocrit	28.2%
RDW	17.5%
White blood cells	99,900/mm^3^
Absolute neutrophil count	71,000/mm^3^
Eosinophil count	2880/mm^3^
Basophil count	20,390/mm^3^
Monocyte count	230/mm^3^
MID#	23,500/mm^3^
Absolute lymphocyte count	5,400/mm^3^
Platelet count	1,69,000/mm^3^

**Figure 1:**
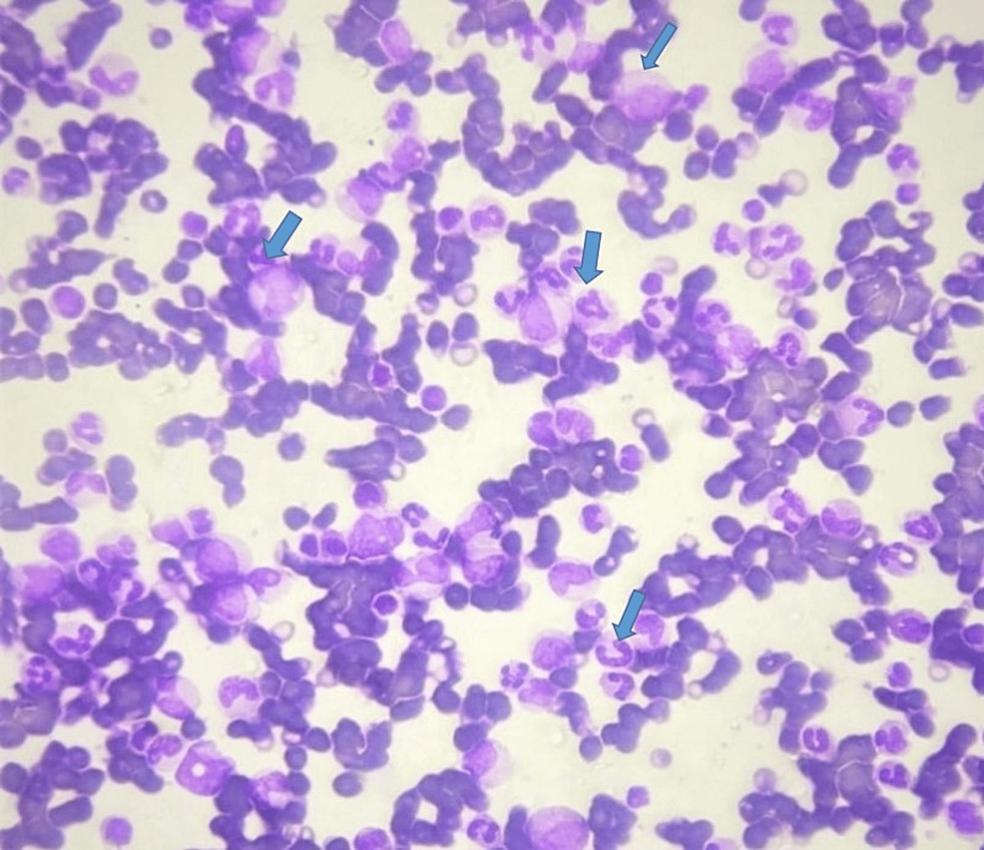
Abundance of immature granulocytes, neutrophils, basophils, and eosinophils (blue arrow) in CML-CP in a Leishman's-stained peripheral smear under 100x magnification. CML-CP: chronic stable phase of chronic myeloid leukaemia

Peripheral smear testing demonstrated the following findings (Figure 1).

Bone marrow aspiration assessment suggested the diagnosis of chronic stable phase of chronic myeloid leukaemia (CML-CP) (Table 2).

**Table 2:** Bone marrow aspiration suggestive of the diagnosis CML-CP. CML-CP: chronic stable phase of chronic myeloid leukaemia

Cellularity	Hypercellular
Erythroid series	Normoblastic maturation seen
Myeloid series	Marked granulocytic hyperplasia with majorly compromising of myelocytes, metamyelocytes, and neutrophils
Myeloid/erythroid (M:E) ratio	8:1
Plasma cells	1% with normal morphology
Differential count: blasts	4%
Promyelocytes	3%
Myelocytes	19%
Metamyelocytes	12%
Neutrophils	55%
Lymphocytes	7%
Eosinophils	6%
Basophils	3%

**Figure 2:**
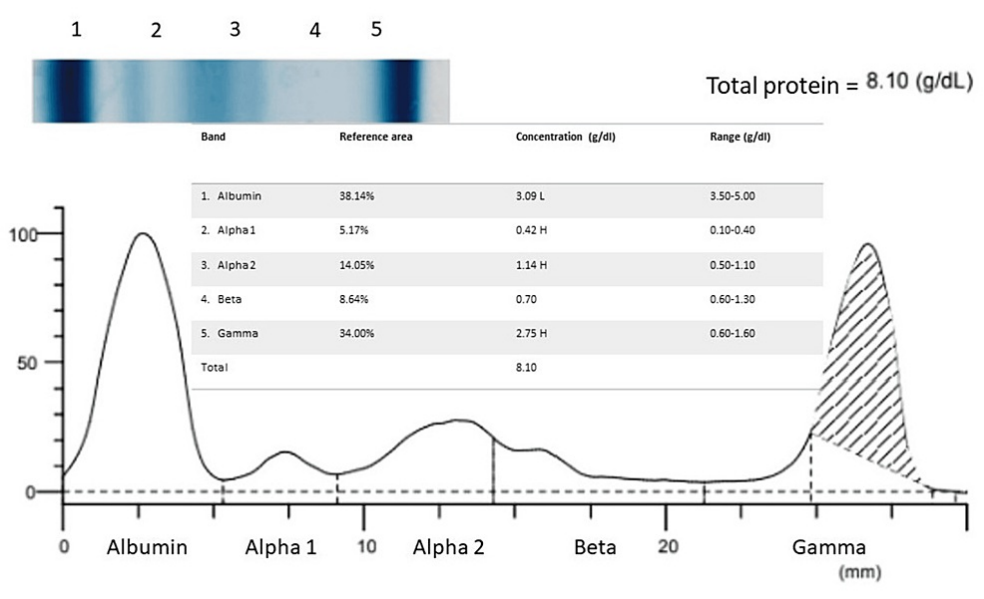
SPEP showed the following findings. SPEP: serum protein electrophoresis

Serum protein electrophoresis (SPEP) depicted the following findings (Figure 2).

The BCR-ABL study was positive, confirming the diagnosis. The Sokal score was 1.02 which suggested intermediate relative risk. The patient was started on standard imatinib mesylate therapy 400 mg once a day to be taken orally daily and was asked to follow up every three months for one year and then six monthly for one year. The patient came for a follow-up after six months. He was asymptomatic. On examination, the spleen and liver were not palpable. Haematological investigations were repeated, which showed the following picture (Table 3). The patient was not willing to do BCR-ABL quantitative polymerase chain reaction (PCR) studies. Imatinib 400 mg once a day was continued.

**Table 3:** Haematological assay of the patient done during follow-up. RDW: red cell distribution width; MID#: mid-range absolute count

Parameters	Result
Haemoglobin	11.3 g/dl
Red blood cells	4.24 million/mm^3^
Haematocrit	35%
RDW	16.3%
White blood cells	7,400/mm^3^
Absolute neutrophil count	5,500/mm^3^
Eosinophil count	296/mm^3^
Basophil count	45/mm^3^
Monocyte count	359/mm^3^
MID#	700/mm^3^
Absolute lymphocyte count	1,200/mm^3^
Platelet count	3,71,000/mm^3^

Further, the patient did not come for follow-up for more than a year as the presenting symptoms subsided. After two years, the patient presented again with complaints of easy fatigability for one month and bone pain which was associated with low-grade fever for 15 days. The patient also gave a history of two to three episodes of syncopal attacks lasting for a few seconds. On general examination, pallor was observed in the lower palpebral conjunctiva and the tip of the tongue. Abdominal examination revealed mild splenomegaly on deep palpation. A haematological assay of the patient was done (Table 4).

**Table 4:** Haematological parameters of the second admission. RDW: red cell distribution width; MCV: mean corpuscular volume

Parameters	Result
Haemoglobin	8.3 g/dl
Red blood cells	3.27 million/mm^3^
Haematocrit	25.1%
RDW	17%
MCV	76.9 fL
White blood cells	76,320/mm^3^
Platelet count	3,02,000/mm^3^

**Figure 3:**
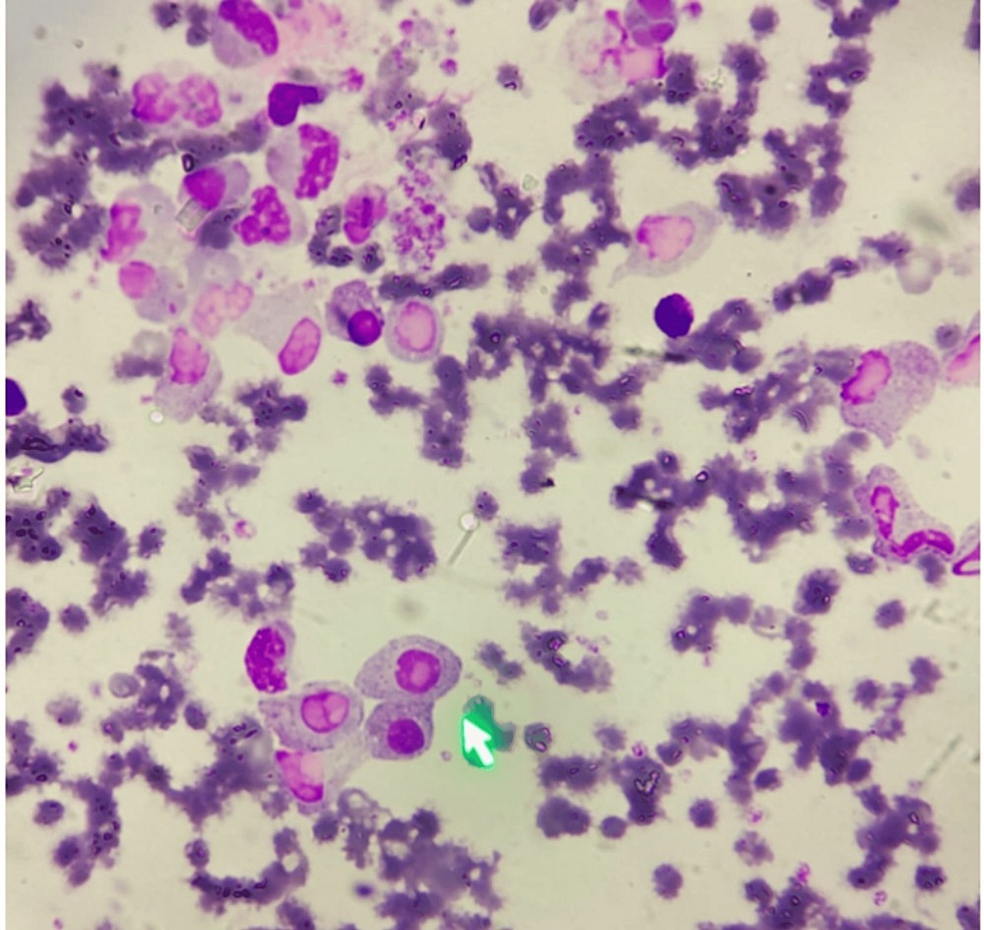
Increased number of plasma cells (more than 20%) (green arrow) in a Leishman's-stained peripheral smear under 100x magnification.

Peripheral smear testing demonstrated the following finding (Figure 3).

**Figure 4:**
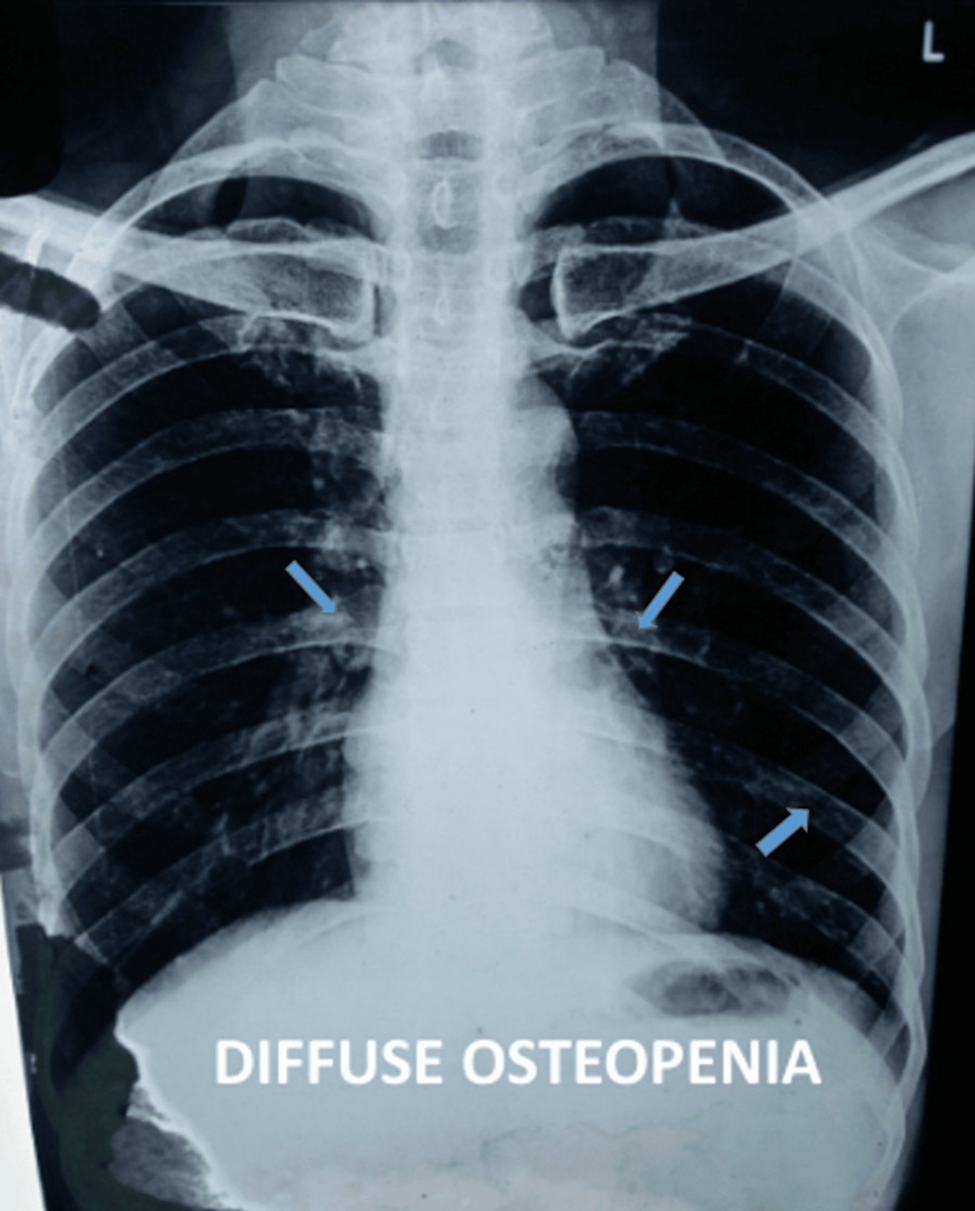
PA view of chest X-ray displaying areas of diffuse osteopenia (blue arrow). PA: posteroanterior

X-ray of the chest was advised as a part of the workup, which displayed diffuse osteopenia (Figure 4).

After careful correlation between all the clinical findings and investigations, a provisional diagnosis of CML progressing into PCL was made. The patient was advised lenalidomide, bortezomib, and dexamethasone therapy for the newly diagnosed PCL. Also, imatinib mesylate was stopped, and dasatinib 100 mg once daily was started. Detailed counselling was advised. The patient was recommended a follow-up of three monthly for six months and six monthly for one year consequently.

## Discussion

CML is a hematopoietic disorder in which a multipotent stem cell acquires an abnormal BCR-ABL fusion gene, which is famously known as the Ph chromosome [1,3,6]. The chromosome comes into existence by a balanced reciprocal translocation between the long arms of chromosomes 9 and 22 (t 9;22) [1,7]. The shortened chromosome 22 is known as the Ph chromosome. This fusion gene has infrequently been identified in essential thrombocytosis, multiple myeloma, and myelodysplastic syndromes [7]. The existence of CML and PCL in a single person is not well understood. Various hypotheses have been put forward, such as since CML arises from a pluripotent stem cell, it may be possible that CML can transform into malignant cells [1,8,9]. This may be detected by next-generation sequencing techniques, fluorescence in situ hybridisation (FISH) analysis, and PCR [3,7].

Another hypothesis indicates that the secondary malignancies developing in a patient with CML may be related to TKI treatment [3]. A study in 2002 conducted by Gunnarsson et al. [1,9] revealed that secondary malignancies such as gastrointestinal tract cancer, breast cancer, prostate cancer, and throat cancer may have been developed due to TKI therapy. It is unclear from the studies that the cause of the development of secondary malignancies is TKI [1,8,10]. However, the TKI and the pathogenesis of CML in combination may play a crucial role in the transformation of CML into PCL [1].

## Conclusions

Synergistic co-occurrence of CML and plasma cell disorder in a single patient is extremely rare in haematological practice. This patient highlights transition from CML to PCL. We presume that TKI and other cytogenetic changes led to this progression. Another potential factor may be the initial gene translocation trigger that led to chronic sustained clonal proliferation. Larger studies into this rare entity may lead to better understanding of the disease process.
